# Pockets of Trouble: Diffuse Jejunal Diverticulosis Causing Refractory Intestinal Microbial Overgrowth

**DOI:** 10.14309/crj.0000000000002003

**Published:** 2026-02-12

**Authors:** Eduardo Cruz, Richard Sukov, Jane Lim

**Affiliations:** 1 Department of Radiology, Cedars-Sinai Medical Center, Los Angeles, CA; 2 Karsh Division of Gastroenterology and Hepatology, Department of Medicine, Cedars-Sinai Medical Center, Los Angeles, CA

**Keywords:** small bowel diverticulosis, small intestinal bacterial overgrowth, intestinal methanogen overgrowth

## CASE REPORT

**Figure 1.**
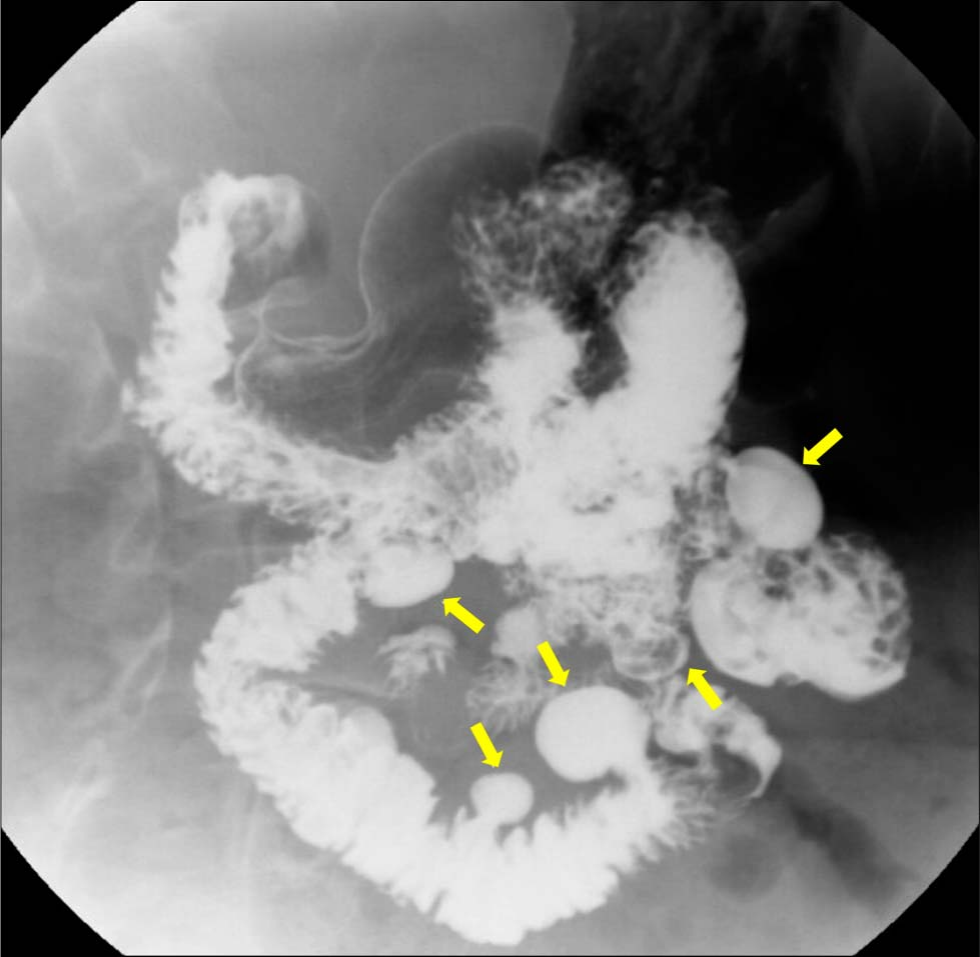
Double-contrast upper gastrointestinal series with small bowel follow-through demonstrating multiple moderate- to large-sized diverticula throughout the jejunum.

**Figure 2.**
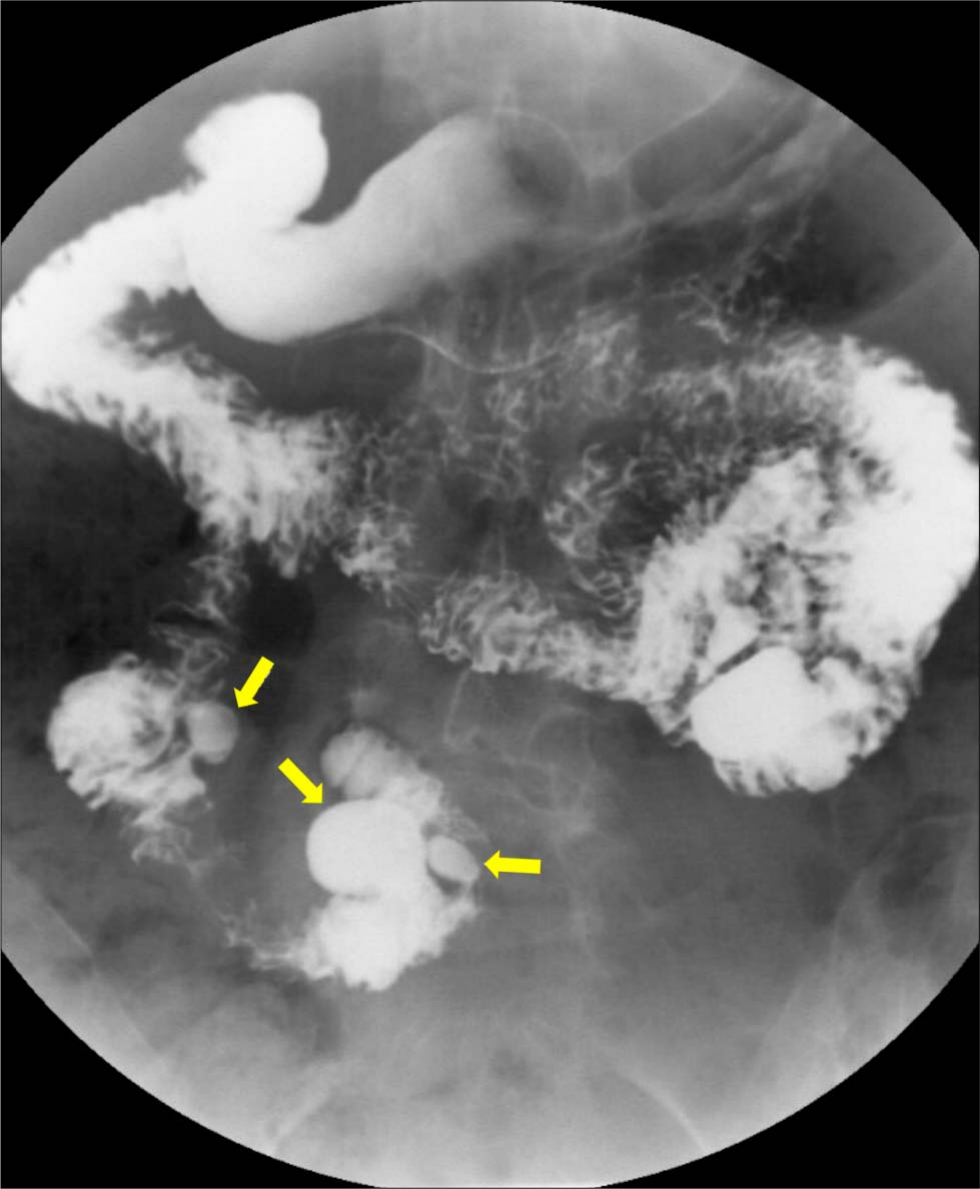
Moderate-to-large sized divertiucla in the jejunum.

A 69-year-old White woman with a body mass index of 23.38 kg/m^2^, chronic constipation, and small bowel diverticulosis complicated by prior diverticulitis presented with refractory small intestinal bacterial overgrowth (SIBO) and intestinal methanogen overgrowth (IMO). Despite four 2-week antibiotic courses of either rifaximin 550 mg 3 times daily or rifaximin plus neomycin 500 mg 2 times daily, symptom relief was short-lived, with bloating and abdominal distension recurring after treatment. Constipation was well controlled with polyethylene glycol. Lactulose breath testing revealed ongoing SIBO and IMO. To assess diverticular burden, a double-contrast upper gastrointestinal series with small bowel follow through was performed, revealing multiple moderate-to-large diverticula throughout the jejunum, a rare location (Figures 1 and 2).^1^ The extensive diverticulosis contributed to refractory SIBO and IMO by promoting intestinal stasis and bacterial fermentation of retained food debris within the diverticula.

A nightly regimen of low-dose erythromycin 50 mg was initiated to augment migrating motor complexes and enhance clearance of the diverticula, leading to significant improvement of symptoms. Small bowel diverticulosis is rare and typically asymptomatic but may lead to refractory intestinal microbial overgrowth.^1–3^ Management options include low-dose prokinetics or when diverticula are localized, and symptom burden is severe, surgical resection.^1,2,4,5^

## DISCLOSURES

Author contributions: E. Cruz drafted the manuscript. R. Sukov provided relevant images. J. Lim and R. Sukov revised the manuscript. All authors approved the submitted version of the manuscript. J. Lim is the article guarantor.

Financial disclosure: None to report.

Informed consent was obtained for this case report.

## ABBREVIATIONS:

IMO: intestinal methanogen overgrowth
SIBO: small intestinal bacterial overgrowth
